# The role of γ-aminobutyric acid and its receptor in metabolic reprogramming and tumor progression

**DOI:** 10.1016/j.bbrep.2026.102611

**Published:** 2026-05-02

**Authors:** Yujie Wang, Junzhao Shao, Ming Wang, Zhenlong Sun, Nan Wang, Wei Li, Hong Luo

**Affiliations:** a College of Medical Laboratory, Dalian Medical University, Dalian, Liaoning, 116044, China; b Medical Laboratory Center, Weihai Municipal Hospital, Cheeloo College of Medicine, Shandong University, Weihai, Shandong, 264200, China; c WEGO Holding Company Limited, Weihai, Shandong, 264200, China

**Keywords:** GABA, GABA receptors, Cancer, Metabolic reprogramming, Tumor microenvironment

## Abstract

Neurotransmitters play fundamental regulatory roles within the central nervous system (CNS), where they modulate neuronal signaling and maintain neural network homeostasis. Beyond their classical functions in the CNS, these signaling molecules can act on other organs throughout the body via vagus nerve terminals, mediating crosstalk between the nervous system and peripheral tissues. Notably, such neurotransmitter-mediated interorgan communication has been implicated in driving metabolic reprogramming, a key adaptive process that can promote tumor initiation and progression. Among the diverse neurotransmitters involved, γ-aminobutyric acid (GABA) has garnered increasing attention, as it exhibits elevated levels in various types of tumors. Accordingly, this review focuses on elucidating the mechanisms of GABA synthesis and secretion, while systematically investigating the role of GABA and its receptors in metabolic reprogramming and the regulation of the tumor immune microenvironment. Collectively, this work aims to unravel the multifaceted contributions of GABA to oncogenesis and thereby to offer novel therapeutic targets for cancer treatment.

## Highlights

• Comprehensive review of GABA's roles in cancer and immunity.
• Synthesis of subtype-specific GABA receptor functions in disease contexts.
• Integrates gut–brain axis: microbiota-derived GABA in neural and tumor regulation.

## 1 Introduction

Neurotransmitters are well-established as key regulators of neural signaling, governing neuronal excitability, synaptic transmission, and central nervous system (CNS) homeostasis [1]. For decades, research has primarily focused on their roles within the CNS, where they orchestrate complex physiological processes ranging from cognition to motor function [[2], [3], [4]]. However, growing evidence has challenged the traditional view of neurotransmitters as “neural-specific” molecules: these signaling mediators can be released from vagus nerve terminals to target peripheral organs, establishing bidirectional communication between the nervous system and peripheral tissues [[5], [6], [7]]. A paradigmatic example of this cross-talk is the gut-brain axis, through which the brain modulates gastrointestinal function by secreting neurotransmitters [8,9], such as γ-aminobutyric acid (GABA) and acetylcholine, via vagus nerve endings, which then bind to corresponding receptors expressed on the gastrointestinal mucosal surface [10]. Beyond the gastrointestinal tract, this interorgan signaling extends to other tissues including the liver [11] and tumor microenvironment [12], enabling neurotransmitters to regulate peripheral physiological processes such as metabolism, inflammation, and tissue repair, thus expanding their functional repertoire beyond neural regulation.

Notably, the dysregulation of neurotransmitter-mediated interorgan signaling has been increasingly linked to metabolic reprogramming [13], and dysregulated metabolic reprogramming serves as a pivotal driver of tumor initiation and progression [14]. Tumor cells exploit this dysregulated metabolic state to rewire their nutrient uptake and energy metabolism, supporting rapid proliferation, invasion, and resistance to therapy. Accumulating studies have demonstrated that neurotransmitters can act as “metabolic modulators” in cancer: by binding to receptors on tumor cells or stromal cells, they trigger signaling pathways that drive key metabolic adaptations such as enhanced glycolysis, glutamine addiction, or lipid synthesis [15,16]. This emerging crosstalk between the nervous system and tumor metabolism has opened a new frontier in cancer biology, highlighting neurotransmitters as potential therapeutic targets beyond traditional oncogenic pathways.

Among the neurotransmitters implicated in tumor-associated metabolic remodeling, GABA has emerged as a particularly intriguing candidate. Classically known as the major inhibitory neurotransmitter in the CNS, GABA is now recognized to be aberrantly upregulated in a wide range of cancers, including colorectal cancer, breast cancer, and glioma [17]. Early studies have linked GABA to tumor progression by regulating cell proliferation, apoptosis, and migration [18]; however, the underlying mechanisms remain incompletely understood. Recent work has shed light on GABA's role in metabolic reprogramming. For instance, GABA signaling has been shown to promote glycolytic metabolism [19] or modulate the metabolic phenotype of immune cells in the tumor microenvironment [20]. Nevertheless, critical questions persist: How are GABA synthesis and secretion dysregulated in tumor cells? How do GABA and receptor subtype-specific signaling cascades separately and synergistically orchestrate tumor metabolic reprogramming and immune modulation to promote tumor progression? Can subtype-selective targeting of GABA receptors serve as a safe and effective therapeutic strategy for cancer?

Given the growing interest in GABA signaling in cancer and the fragmented nature of current evidence, this review summarizes research findings on GABA synthesis and secretion mechanisms in tumor cells, GABA receptor-mediated metabolic reprogramming, and interactions between GABA signaling and the tumor immune microenvironment. Through this discussion, we aim to explore GABA's potential implications for cancer treatment.

## 2 Molecular basis of GABA synthesis and degradation

**Fig. 1:**
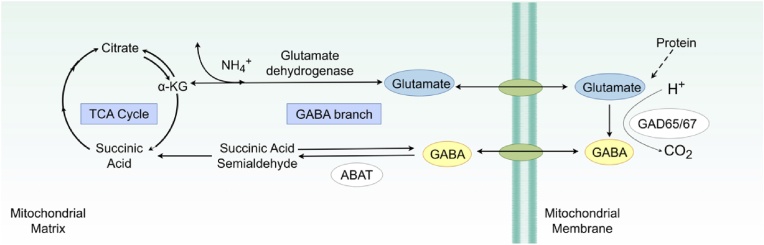
**GABAergic synapse and the mitochondrial GABA shunt.** Shows the synthesis of GABA from glutamate by GAD65/67, its catabolism by ABAT/GABA-T, and the interaction with mitochondrial TCA cycle metabolism. The diagram was drawn by figdraw.com. The copyright ID is TSTRU21222.

GABA is an important inhibitory neurotransmitter in the central nervous system. Beyond its synthesis in the nervous system, GABA can also be produced by tumor cells and immune cells, especially Tumor-infiltrating B cells (TIL-B). Within the mitochondrial matrix, glutamate derived from precursors such as glutamine or glucose undergoes decarboxylation catalyzed by glutamate decarboxylases (GAD65 and GAD67), leading to the production of GABA as part of the GABA branch. [21,22]. GAD65 primarily participates in GABAergic synaptic transmission and plasticity [23], while GAD67 regulates metabolic GABA synthesis [24]. For GABA catabolism, γ-aminobutyrate transaminase (ABAT; also referred to as GABA-T) catalyzes the transamination of GABA with α-ketoglutarate (α-KG), yielding succinic semialdehyde (SSA) and l-glutamate. SSA is then funneled into the tricarboxylic acid (TCA) cycle, where it is further oxidized to succinic acid—a metabolite that feeds into ATP-generating pathways (Fig. 1) [25]. This metabolic cascade is associated with the release of H^+^ and NH_4_^+^, while CO_2_ is produced as a byproduct of subsequent TCA cycle reactions.

## 3 Non-vesicular GABA release mechanisms in the tumor microenvironment

The maintenance of extracellular GABA concentrations relies not only on enzymatic synthesis but may also on non-vesicular release mechanisms, including regulation by the GABA transporter (GAT) transporter, the Best1 ion channel, and tumor cytokine-mediated GABA release [26].

GATs are Na^+^/Cl^−^-dependent GABA transporters with four subtypes (GAT-1/2/3/4). GAT-1 predominates in neurons for GABA uptake, while GAT-2/3/4 are expressed in peripheral tissues and can mediate GABA release under depolarization [27]. Their activity is regulated by endocytosis and calcium signaling. In cerebellar granule cells (Bergmann glia), GAT-1 is capable of releasing sufficient quantities of GABA under depolarizing conditions to activate surrounding GABA receptors. This reverse transport typically occurs when intracellular Na^+^ concentrations are elevated or when the membrane potential is depolarized [28]. In the tumor microenvironment, if high K^+^ or altered ion gradients exist, GAT-1 could theoretically undergo reverse transport, but experimental evidence supporting this remains limited. In epithelial cellular models, GAT-2 and GAT-4 have also been shown to mediate reverse GABA transport under high K^+^ or high Na^+^ conditions [29].

In contrast, GABA in the tumor microenvironment is primarily derived from tumor cells themselves or tumor-associated astrocytes, and the GABA release mechanism in these cells tends to involve the interaction between inflammatory cytokines and the bestrophin-1 (Best1) channel. Best1 is a calcium-activated chloride channel confirmed to mediate non-vesicular GABA release from astrocytes in murine models, generating tonic inhibitory currents that regulate extracellular GABA levels [30,31]. Recent studies in HEK293 and iPSC-RPE cells shows that GAD65 may directly interacts with Best1, enhancing both Cl^−^ currents and GABA permeability. Extracellular GABA also activates Best1 at nanomolar concentrations, indicating Best1 functions might as a GABA receptor [32].

In vitro and mouse model studies report that inflammatory cytokines IL-1β can significantly induce elevated intracellular Ca^2+^ levels in astrocytes, triggering Best1 channel opening and thereby releasing GABA through non-vesicular pathways [[33], [34], [35]], which may play an important role in the tumor immune microenvironment. Other pro-inflammatory cytokines beyond IL-1β, including TNF-α, exert similar effects; TNF-α released in inflammatory CNS diseases alters astrocytic Ca^2+^ homeostasis and membrane potential, enhancing intracellular Ca^2+^ or glutamate-induced Ca^2+^ responses [36]. Since elevated intracellular Ca^2+^ contributes to Best1 channel opening [37] or affects GAT-1 activity [32,34,37] we infer that TNF-α may drive non-vesicular GABA release by regulating astrocytic Ca^2+^ signaling pathways. However, there is currently no direct experimental evidence supporting this inference, which provides a new direction for future research.

In addition, studies have identified a monocyte subpopulation with high BEST1 expression in the peripheral blood of head and neck squamous cell carcinoma patients, revealing that tumor cytokines can promote increased BEST1 expression in monocytes, thereby enhancing their pro-tumorigenic functions [38]. Collectively, these studies suggest that inflammatory cytokine-induced, Best1-mediated GABA release may also play a promotive role in the inflammatory microenvironment of tumors.

## 4 Dysregulated expression of enzymes involved in GABA synthesis and degradation in tumor

The biosynthesis and catabolism of GABA are tightly regulated by key enzymes under physiological conditions. GAD65, GAD67 and ABAT serve as the rate-limiting enzyme in GABA synthesis and degradation via the mitochondrial GABA shunt. However, in the nutrient-depleted, poorly vascularized tumor microenvironment, cancer cells undergo extensive metabolic reprogramming to sustain aberrant proliferation, invasion, and survival—an adaptive hallmark that frequently disrupts the homeostatic expression of enzymes involved in GABA metabolism [39]. Accumulating animal and clinical data reveal that dysregulated GAD family members and ABAT expression is a conserved molecular feature across multiple cancers, disrupting GABA synthesis-degradation balance, causing intratumoral GABA accumulation and promoting tumor progression [40].

Correlative studies suggest GAD67 is upregulated in various tumors and may contributes to cancer progression, while GAD65 involvement in common epithelial cancers remains largely unexplored [41]. However, recent studies using immunohistochemical (IHC) staining analysis of patient tissue samples indicate that GAD65 (encoded by GAD2) serves as a highly specific marker for pancreatic neuroendocrine neoplasms, suggesting tissue-of-origin–dependent expression patterns [42]. In lung and colon cancer cells utilize the abundant glutamine in the tumor environment for metabolism and GABA synthesis by abnormally expressing the glutamate decarboxylase family member GAD67 [17]. Overall, available evidence indicates that GAD67 potentially facilitates tumor progression, as its overexpression in tumor tissues has been associated with enhanced proliferative and invasive potential in multiple cancer types [[43], [44], [45]]. Conversely, as the GABA catabolic enzyme, ABAT expression is downregulated in breast cancer, liver cancer, and renal cancer cells according to some studies [46]. Clinical sample analyses reveal that in breast and renal cell carcinomas, high GAD67 expression with reduced ABAT levels impairs GABA degradation, accelerating intratumoral GABA accumulation. Consistently, ectopic ABAT expression reduces GABA levels and attenuates cancer cell proliferation and migration [46,47].

## 5 Bidirectional regulation by GABA branch metabolites in cancer

**Fig. 2:**
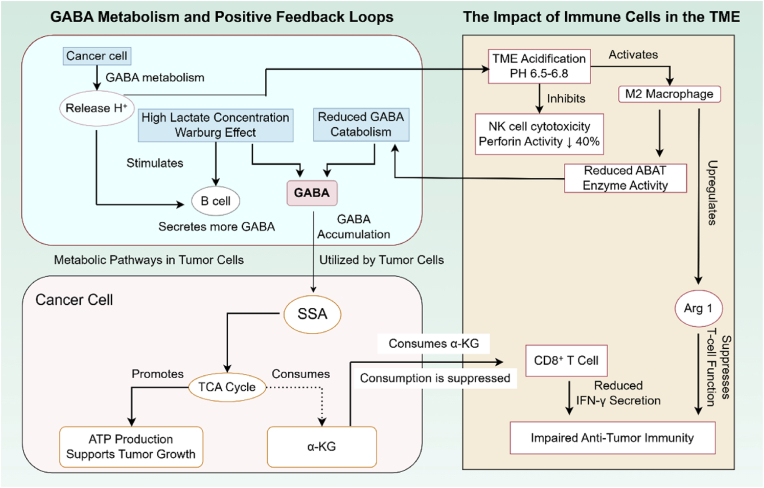
**GABA metabolism drives tumor immune escape via metabolic and epigenetic reprogramming.** In cancer cells, GABA is metabolized via the glutamine-GABA axis, using α-KG for the TCA cycle and ATP production, which depletes α-KG, hinders T cell function, and reduces IFN-γ production. The resulting H^+^ release acidifies the tumor microenvironment, reducing NK cell effectiveness and encouraging M2 macrophage polarization. Additionally, GABA secretion from B cells and decreased ABAT activity in macrophages create a self-reinforcing immunosuppressive loop. The diagram was drawn by figdraw.com. The copyright ID is UYSWU538e1.

The GABA metabolic pathway has emerged as a key regulator of tumor progression, with its branch metabolites exerting bidirectional effects on both tumor cell bioenergetics and the immunomodulatory landscape of the TME (see Fig. 2). GABA metabolism contributes to tumor progression through three interconnected mechanistic axes: (1) TCA cycle flux and bioenergetic support, (2) α-KG depletion and epigenetic modulation, and (3) TME acidification and immunosuppression.

### 5.1 TCA cycle flux and bioenergetic support

Succinic semialdehyde (SSA), a critical intermediate in GABA catabolism, can be shuttled into the tricarboxylic acid (TCA) cycle to drive ATP production. This metabolic route provides an alternative energy source that may support tumor cell bioenergetics under nutrient-limiting conditions. In vitro and mouse model studies in lung cancer suggest that SSA-derived TCA cycle entry reportedly contributes approximately 15% of the total energy requirements in lung cancer cells [48]. These findings indicate that GABA catabolism may serve as a metabolic fuel source in certain tumor contexts.

### 5.2 α-KG depletion and epigenetic modulation

GABA metabolism induces depletion of α-ketoglutarate (α-KG), a key cofactor for epigenetic modifiers including TET family DNA demethylases and JmjC domain-containing histone demethylases. α-KG depletion may inhibit these enzymes, leading to altered DNA and histone methylation patterns that could affect immune cell function and tumor cell gene expression. Analyses of TCGA data, hepatocellular carcinoma cell lines, and mouse models revealed that GABA metabolism induces depletion of α-KG, a key cofactor for epigenetic modifiers, which may inhibit CD8^+^ T cell epigenetic reprogramming and impair interferon-gamma (IFN-γ) secretion [49]. This mechanism suggests a metabolic basis for GABA-mediated T cell dysfunction.

### 5.3 TME acidification and immunosuppression mechanism

Notably, GABA metabolic processes are associated with H^+^ release, contributing to TME acidification (pH ∼6.5–6.8). An acidic microenvironment may impair immune cell function through multiple mechanisms, including reduced cytotoxic activity and altered macrophage polarization. In pancreatic ductal adenocarcinoma, analyses of TCGA/GEO datasets, PDAC cell lines, and xenograft models linked this acidic microenvironment to immunosuppressive effects, including reduced NK cell cytotoxic activity and enhanced arginase 1 (Arg1) activation in M2-polarized macrophages [50]. These observations suggest that GABA-driven acidification may contribute to immune evasion in specific tumor types.

Collectively, GABA-mediated metabolic reprogramming, encompassing altered tumor cell bioenergetics (TCA flux), epigenetic modulation (α-KG depletion), and TME acidification, may create conditions that facilitate tumor immune evasion. These metabolite-driven effects operate in parallel with receptor-mediated signaling mechanisms, as described in the following section.

## 6 GABA receptor expression and oncogenic signaling axes

### 6.1 GABA receptor classification and structural features

GABA functions as a secreted transmitter that transduces signals from cell to cell by binding to membrane bound GABA receptors [51]. There are two main classes of GABA receptors: GABA_A_ receptors are ligand-gated ion channels (ionotropic receptors), whereas GABA_B_ receptors are G protein-coupled receptors (metabotropic receptors) [52]. Among them, the GABA_A_ receptor is the predominant type, belonging to the ligand-gated chloride channel receptor family, composed of a pentameric structure formed by five subunits. These subunits are encoded by 19 distinct genes, which are categorized into 8 subclasses according to sequence homology. These subunit classes comprise α1–6, β1–3, γ1–3, δ, ε, π, θ, and ρ1–3, which are encoded by the genes *GABRA1–6, GABRB1–3, GABRG1–3, GABRD, GABRE, GABRP, GABRQ, and GABRR1–3*, respectively. These subunits can combine as home- or heteromers. [53,54]. The GABA_B_ receptor is a heterodimeric G-protein coupled receptor composed of GABBR1 and GABBR2, both of which are generally required for receptor function [55]. Through alternative splicing, the *GABBR1* gene produces a variety of transcript variants, the most prominent of which are GABBR1a and GABBR1b. Studies in cultured neurons in vitro have shown that upon activation, GABA_B_ receptor may regulates Ca^2+^ and K^+^ channels [56,57]. The following Table 1 presents the classification of GABA receptors.

**Table 1:** Classification of GABA receptors.

Category	GABA_A_ Receptor	GABA_B_ Receptor	GABA_C_ Receptor (GABA_A_-ρ)
**Structural Features**	Pentameric ion channel (Subunits: GABRA1-6, GABRB1-3, GABRG1-3, GABRD, GABRE, GABRP, GABRQ.) [54]	Heterodimeric GPCR (GABBR1a/b and GABBR2) [58]	Homomeric, heteromeric GABRR1-3 [59]
**Signaling Mechanism**	Ligand-gated Cl^−^ influx → Neuronal hyperpolarization [60]	Gi/o-coupled: • Postsynaptic: ↑K^+^ efflux [57] • Presynaptic: ↓Ca^2+^ influx→ Reduced [56] neurotransmitter release	Ligand-gated Cl^−^ channel (Bicuculline/Baclofen insensitive) [61]
**Key Subunit Functions**	• GABRA1: Sedation • GABRA2: Anxiolysis • GABRA5: Memory modulation [62]	• GABBR1a: Targets glutamatergic terminals • GABBR1b: Targets GABAergic dendrites [58]	• GABRR1 subunits: Retina-specific Cl^−^ conductance [63]
**Pathological Relevance**	• GABRA6 subunit mutations: Epilepsy/Anxiety [64] • ↑Expression in prostate cancer [65]	• ↓Expression in colorectal cancer [66] • Addiction pathway modulation [58]	• Retinal degenerative disorders [67]

### 6.2 Cancer-specific expression patterns

Accumulating evidence suggests that GABA receptors particularly GABA_A_ receptors may exhibit expression patterns associated with tumor proliferation, migration, and immune evasion across multiple cancer types. [18,43,48]. GABA receptor subunit expression varies across cancer types, with subtype-specific functions that could contribute to disease progression.

**Fig. 3:**
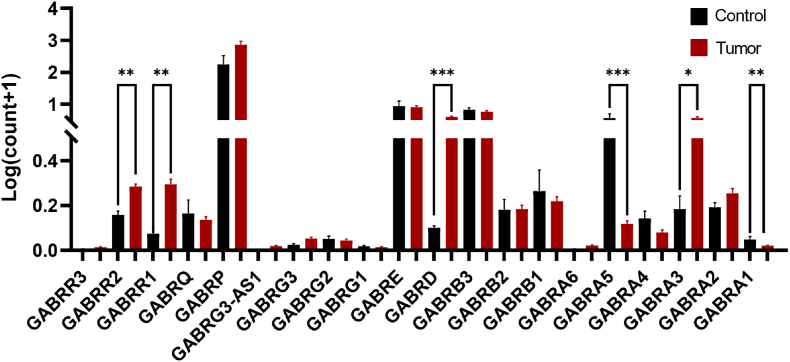
**Analysis of the Expression of Different GABA Receptor Subunits in Gastric Cancer.** Raw mRNA sequencing count data of GABA receptor subunits in gastric tumors were obtained from TCGA via the UCSC Xena Data Portal (http://xena.ucsc.edu/). Differential expression analysis revealed that GABBR1 (*p* = 0.0066), GABBR2 (*p* = 0.0017), GABRD (*p* = 0.0001), and GABRA3 (*p* = 0.0177) were significantly upregulated, while GABRA5 (*p*= 0.0001) was markedly downregulated in gastric cancer tissues compared to normal controls. No statistically significant differences were observed for other tested subunits (∗*p < *0.05, ∗∗*p < *0.01, ∗∗∗p < 0.001).

In breast cancer, in vitro studies demonstrate that elevated GABRA3 expression may activate the AKT pathway, potentially contributing to increased metastatic capacity. Meanwhile, edited GABRA3 isoforms appear to counteract some oncogenic effects of wild-type GABRA3. This result suggesting mRNA editing could serve as a potential prognostic biomarker [58]. GABRP may promote migration of basal-like breast cancer cells, possibly involving ERK1/2 pathway activation [59]. In addition, GABA_A_ receptor signaling plays a key role in the invasion and metastasis of triple-negative breast cancer (TNBC), suggesting that this subtype of cancer may benefit from treatment strategies targeting GABA receptors. In glioma, dysregulated GABA_A_ receptor subunit expression modulates tumor cell proliferation and stemness, positioning these receptors as one of the few druggable targets in nervous system tumors and garnering significant therapeutic interest [60]. In lung cancer patients, altered GABA receptor subunit expression profiles appear to correlate with clinical outcomes in non-small cell lung cancer, hinting at subtype-specific relevance of GABAergic signaling [61]. In patients with colorectal adenocarcinoma, increased expression of GABRD also predicts poor prognosis [62] and GABBR1 has been associated with malignant phenotypes in colorectal cancer patients and tissue samples [63]. Our analysis of gastric cancer tissues revealed distinct expression of GABA receptors between tumor and control tissues (Fig. 3). The expression levels of GABBR1, GABBR2, GABRD and GABRA3 were significantly upregulated, whereas GABRA5 was markedly downregulated.

GABA receptors not only play a key role in tumor cells but also exert significant functions in immune cells, potentially contributing to immunosuppressive effects. These effects appear to involve regulation of various immune cell subsets within the tumor microenvironment and peripheral immune system. These changes are largely consistent with observations reported in other cancer types. Additional studies have reported GABA receptor alterations in other malignancies, including increased GABAA receptor expression in prostate cancer [64] and decreased expression in colorectal cancer [65], suggesting context-dependent functions across tumor types.

### 6.3 Receptor-mediated oncogenic signaling pathways

Building on the expression patterns described above, we now elaborate on the key signaling pathways directly modulated by GABAergic receptor activation to drive cancer progression.

*AKT Pathway:* Cell-based experiments and in vivo mouse suggest that GABRA3 upregulation may activate the AKT pathway to enhance metastatic potential, although the mRNA-edited form of GABRA3 suppresses GABRA3-mediated Akt activation and breast cancer metastasis [58,66,67]This mechanism has been primarily characterized in breast cancer models.

*ERK Pathway:* GABRP-HER2 complex formation may enhance ERK1/2 phosphorylation and contribute to trastuzumab resistance through elevation in the half-maximal inhibitory concentration [59]. This interaction may amplify oncogenic signaling with implications for tumor progression [68].

*β-catenin Pathway:* GABAB receptor signaling via Gi/o proteins may inhibits the adenylate cyclase-cAMP-PKA axis, potentially relieving GSK-3β-mediated suppression of β-catenin phosphorylation [17]. This mechanism may promote proliferation and invasion in cancers expressing GABA_B_ receptors.

*YAP/TAZ Pathway:* Activation of the GABA_A_ receptor assembled from GABRA5, GABRB3, and GABRG subunits triggers calcium-dependent Calpain-mediated cleavage of AMOTL2. This may lead to the release of YAP/TAZ, upregulation of CTGF and CYR61 expression, and ultimately accelerates lung metastasis [68].

*STAT3 Pathway:* Preclinical mouse models show that GABA can promote T cell exhaustion through upregulating PD-L1 via GABA_A_ receptor-activated STAT3 signaling [17]. In non-small cell lung cancer, clinical evidence indicates that GABA_B_ receptor signaling may be associated with phosphorylates STAT3 in macrophages, potentially elevating IL-10 production and suppressing dendritic cell maturation by reducing CD80/CD86 expression and expand regulatory T cell populations compared to adjacent parenchyma [61].

*NF-κB Pathway:* In pancreatic adenocarcinoma, patient tissue samples indicate that GABRP-mediated NF-κB activation in stellate cells may induce CCL2 secretion, potentially recruiting tumor-associated macrophages [60]. This process may contribute to TGF-β1-driven fibrosis and increased collagen deposition.

*Additional Mechanisms:* In colorectal cancer patients, GABRD overexpression has been associated with poor prognosis [62]. Furthermore, GABBR1 has been shown to promote proliferation and invasion through targeted regulation by miRNAs including miR-106a/b, miR-20a/b, and miR-17 in colorectal cancer [69]. Tumor vascularization may be potentiated via GABA-mediated activation of endothelial receptors, possibly affecting VEGF-dependent angiogenesis [48]. In vitro studies in breast cancer models demonstrate that GABA_A_ receptor activation can trigger Cl^−^ influx, inducing membrane hyperpolarization that may suppress apoptosis through voltage-gated calcium channel-dependent pathways [60].

Given the conserved expression patterns across cancer types, we hypothesize that similar downstream signaling mechanisms may operate in gastric cancer: GABRA3 upregulation may activate the AKT pathway; GABRD overexpression may confer poor prognosis through mechanisms analogous to colorectal adenocarcinoma; and GABBR1/GABBR2 upregulation may promote proliferation through miRNA-mediated regulation. However, these mechanistic links in gastric cancer remain to be experimentally validated.

### 6.4 Receptor functions in immune cells

GABA receptors not only play a key role in tumor cells but also exert significant functions in immune cells, potentially contributing to immunosuppressive effects within the tumor microenvironment.

*Treg Cell Activation:* In vitro co-culture models of liver cancer and immune cells suggest that GABA_A_ receptor subunits such as GABRA and GABRB on regulatory T (Treg) cells may enhance immunosuppressive activity and potentially inhibit CD8^+^ T cell cytotoxicity, possibly favoring tumor progression. [49]. Additionally, the activation of GABA receptors in vitro has been shown to impair the anti-tumor potential of peripheral blood mononuclear cells (PBMCs), a population that includes key effector immune cells involved in anti-tumor responses [70]. GABA treatment may suppress cytotoxic activity, potentially through reduced granzyme B secretion and perforin downregulation via GABAB receptor-mediated cAMP pathway inhibition [60].

*Macrophage and MDSC Modulation:* Furthermore, in female glioblastoma (GBM) patients and mouse models, GABA and its analogs may modulate the metabolic profile of granulocytic myeloid-derived suppressor cells (gMDSCs), potentially dampening their immune surveillance function within the tumor microenvironment [71]. In non-small cell lung cancer, GABAB receptor signaling may suppress dendritic cell maturation by reducing CD80/CD86 expression and expand regulatory T cell populations [61].

Collectively, these findings suggest that GABA receptors may contribute to immunosuppressive signaling across multiple immune cell types, potentially creating conditions that could support tumor growth in certain contexts.

### 6.5 Tissue specificity and therapeutic considerations

**Fig. 4:**
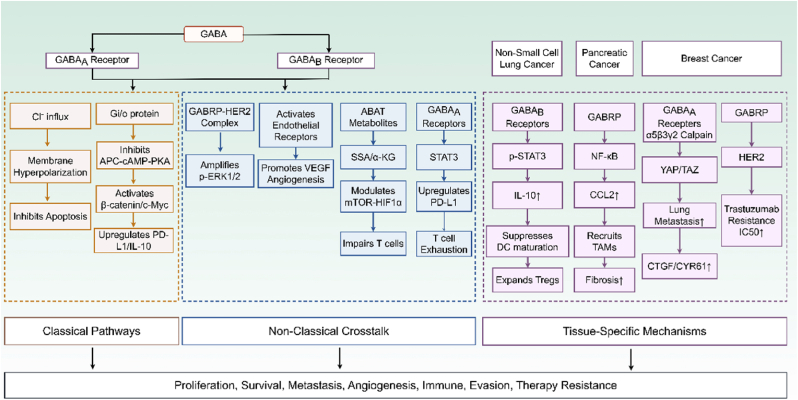
**Diverse oncogenic signaling pathways mediated by GABA receptors across cancer types.** GABA drives tumor progression through classical ion channel-dependent pathways (Cl^−^ influx, cAMP inhibition) and non-classical signaling crosstalk (e.g., HER2 complex, angiogenic activation). It facilitates immune evasion by inducing PD-L1, IL-10, and T cell exhaustion. Tissue-specific mechanisms, such as YAP/TAZ activation in breast cancer, NF-κB/CCL2 in pancreatic cancer, and STAT3/IL-10 in lung cancer, highlight the context-dependent rewiring of the tumor microenvironment by GABAergic signaling. The diagram was drawn by figdraw.com. The copyright ID is WWWPS8201a.

Although most studies have defined GABA receptor subunit as an oncogene, it is noteworthy that the GABA signaling pathway exhibits high tissue specificity in its functions. In specific neuroendocrine tumors or under certain microenvironmental conditions, GABA signaling may exhibit tumor-suppressive properties. Since GABR subunit is predominantly expressed in the central nervous system, drugs targeting this receptor may need to address the issue of blood-brain barrier permeability or face the risk of neurotoxicity. These considerations have important implications for therapeutic development. As shown in Fig. 4, the pro-tumorigenic signals derived from GABA receptors are not restricted to cancer cells themselves; instead, they remodel the TME through multiple mechanisms, thereby establishing an immunosuppressive niche that enables tumors to evade immune surveillance and elimination.

In summary, GABA receptor signaling constitutes a critical oncogenic axis parallel to metabolic reprogramming. Beyond these cell-intrinsic mechanisms, emerging evidence indicates that GABA also remodels the tumor immune microenvironment to facilitate immune evasion, as discussed in the following section.

## 7 Remodeling of the tumor immune microenvironment by GABA

**Fig. 5:**
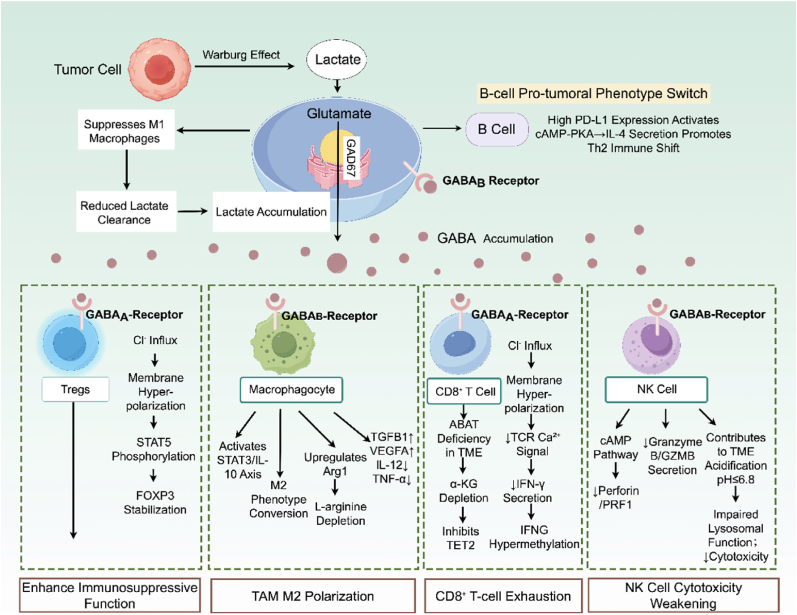
**Multi-mechanistic network of GABAergic immunosuppression in the TME.** GABA from tumor and B cells suppresses CD8^+^ T and NK cell activity through Cl^−^ influx and metabolic-epigenetic changes, while boosting immunosuppression by activating Tregs, polarizing macrophages to M2, and altering B cells to support tumors. A lactate-GABA feedback loop and acidic tumor environment strengthen this suppression. The diagram was drawn by figdraw.com. The copyright ID is UYSWU538e1.

TME is a complex system of tumor, immune, and stromal cells, along with soluble factors [72]. Beyond direct receptor-mediated signaling, GABA exerts secondary effects that remodel the TME through metabolic-immune crosstalk and intercellular communication loops (Fig. 5) [73].

### 7.1 Metabolic-immune crosstalk

GABA metabolism intersects with immune cell function through multiple metabolite-dependent mechanisms. ABAT deficiency may impair GABA catabolism, potentially depleting α-KG and affecting TET2-mediated IFNG demethylation in CD8^+^ T cells [49]. This metabolic constraint could limit effector T cell function independent of receptor signaling. GABA-induced microenvironment acidification could also impair NK cell lysosomal function [74]. This effect stems from metabolite accumulation rather than receptor activation, representing a distinct mechanistic axis. Clinically, intratumoral GABA levels appear negatively correlated with CD8^+^ T cell infiltration in liver cancer [75], suggesting that metabolic factors may shape immune cell distribution within the TME.

### 7.2 Cell-cell communication loops

Emerging evidence indicates that GABA mediates intercellular communication within the TME through paracrine and autocrine loops.

Lactate-GABA Feedback: Tumor-derived lactate may upregulate GAD67 in B cells, promoting GABA synthesis, which could suppress M1 macrophages via GABA_B_ receptors and potentially reduce lactate clearance [20]. This positive feedback loop may amplify immunosuppression.

*B Cell-Treg Axis:* Tumor-infiltrating B cells (TIL-B) synthesize GABA via GAD67, forming an autocrine loop. Studies found that GABA^+^ B cells highly express PD-L1 and, through GABAB receptor activation of the cAMP-PKA pathway, induce IL-4 secretion, promoting a Th2-type immune shift [20,76]. In pancreatic cancer patients, the proportion of GABA^+^ B cells positively correlate with Treg infiltration, and patients with a high proportion have shortened overall survival [76].

*Macrophage-T Cell Suppression:* In a lung cancer mouse model, tumor-derived GABA increased the proportion of M2 marker CD206^+^ macrophages while upregulating arginase (Arg1) activity, leading to l-arginine depletion in the microenvironment and inhibiting T cell mitochondrial function [20,48]. Single-cell RNA sequencing of human lung cancer tissues revealed significant enrichment of immunosuppressive genes such as TGFB1, VEGFA and downregulation of inflammatory cytokines such as IL-12, TNF-α in GABA-treated TAMs [48].

### 7.3 Cancer-specific immune remodeling

The immunomodulatory effects of GABA exhibit cancer-type specificity. In gastric cancer, patients with high GABBR1 expression exhibit a 60% reduction in tumor tissue tryptophan concentration, which correlates with T cell exhaustion markers like TIM-3 and LAG-3 [77]. This metabolic-immune interaction may represent a distinct mechanism of immune evasion in gastric cancer. In the liver cancer, in vitro experiments suggest that GABA concentration in microenvironment positively correlates with Treg infiltration, while ABAT overexpression could reduce the Treg proportion and restore CD8^+^ T cell function [49]. In Lung Cancer, GABAB receptor signaling may suppress dendritic cell maturation by reducing CD80/CD86 expression and expand regulatory T cell populations compared to adjacent parenchyma [61]. These cancer-specific patterns highlight the context-dependent nature of GABA-mediated immune remodeling.

### 7.4 Therapeutic implications

Targeting GABA metabolic enzymes, such as GAD67 inhibitors, or receptors, like GABA_B_ receptors antagonists, offers potential for reversing immunosuppression and developing new combination immunotherapy strategies. Future research should explore the heterogeneity of GABA's immunomodulatory roles across cancer types and create specific delivery systems to minimize neurotoxicity.

In summary, GABA remodels the TME through secondary effects involving metabolic-immune crosstalk and intercellular communication. These mechanisms, together with the metabolic and signaling axes described in Sections 5, 5.1, constitute a comprehensive framework for understanding GABA's pro-tumor functions.

## 8 Perspective

Growing evidence links GABA signaling to cancer progression. This review summarizes recent findings on GABA expression, metabolism, and immune interactions, outlining a potential framework involving metabolite-driven changes, receptor-mediated signaling, and immune remodeling. Specifically, this includes TCA flux alterations, subtype-specific cascades, and metabolic-immune crosstalk. While this suggests GABA may coordinate metabolic and immunomodulatory functions, most insights remain preclinical. Further validation in human tumors and clarification of tissue-specific effects is needed to support clinical translation.

Firstly, inconsistencies exist regarding the expression patterns and tissue specificity of GABA synthetases, particularly GAD family members, in tumors. GAD65 is primarily responsible for activity-dependent GABA synthesis, and is particularly involved in GABA loading into synaptic vesicles and rapid release [78]. GAD65 is more preferentially expressed in endocrine tissues or tissues regulated by hormones/neurotransmitters, and is more significantly affected by estrogen [79] and histone modifications [78]. Notably, recent studies have demonstrated that GAD65 serves as a highly specific marker for pancreatic neuroendocrine neoplasms, suggesting it may play an important role in tumors arising from endocrine lineages [42]. In contrast, GAD67 is more strongly influenced by DNA methylation [80] and *Wnt* signaling [81], and is more prone to be upregulated in metastatic tumors or those with high metabolic demand. This may account for the higher expression of GAD67 in tumor. However, the differential expression patterns of GAD65 versus GAD67 across tumor types may reflect tissue-of-origin effects, and this distinction warrants further exploration in future research.

Additionally, the functions of GABA receptor subunits, especially GABA_A_ receptors composed of multiple subunits, exhibit high tissue and cancer-type specificity, leading to considerable complexity and contradictions. For example, in colorectal cancer, GABRD is the only significantly upregulated receptor subunit associated with poor prognosis [82], while GABRA3 promotes metastasis but its RNA-edited isoforms exert an inhibitory effect on this process [58], suggesting that different variants of the same gene may have opposing roles, whose regulatory networks and clinical implications are not fully elucidated. Furthermore, questions persist regarding whether GABA signaling possesses tumor-suppressive properties under specific conditions.

The immunomodulatory mechanisms mediated by GABA are described as multi-layered, yet the relationships between these mechanisms are insufficiently explored. For instance, GABA-induced inhibition of CD8^+^ T cells involves both ion channel-dependent membrane hyperpolarization [83] and metabolic-epigenetic interference caused by ABAT deficiency [84], but it remains unclear whether these two mechanisms occur simultaneously, have a hierarchical order, or predominate under different TME conditions.

Another critical gap lies in the incomplete understanding of GABA regulatory mechanisms within the TME. First, the specific contribution of non-vesicular release (via Best1 channels or GAT reverse transport) in tumors remains underexplored; clarifying these pathways could offer strategies to block local GABA accumulation without disrupting CNS homeostasis. Second, while a lactate-GABA positive feedback loop involving tumor cells, B cells, and macrophages is established, the molecular sensors linking lactate to GABA metabolism are undefined. For instance, how lactate upregulates GAD67 in B cells or suppresses ABAT in M2 macrophages is unclear. Emerging evidence suggests several candidate pathways: GPR81 (HCAR1), a high-affinity lactate receptor expressed on immune cells [85,86], inhibits adenylate cyclase and reduces cAMP levels upon activation, potentially modulating PKA-dependent transcriptional programs [87]. Recent work shows that lactate-GPR81 signaling promotes regulatory T cell function and suppresses effector T cell responses in the tumor microenvironment [86], suggesting analogous mechanisms may operate in B cells. Beyond membrane receptors, lactate can stabilize HIF-1α by inhibiting prolyl hydroxylase activity and activate mTORC1 signaling [88], both of which are involved in metabolic reprogramming and gene regulation in immune cells [89]. However, whether these pathways directly regulate GAD67 expression in tumor-infiltrating B cells remains to be experimentally validated. Investigating the specific lactate sensors and downstream effectors governing GAD67 expression in tumor-infiltrating B cells represents a critical area for future research. Understanding these pathways may reveal therapeutic opportunities to disrupt lactate-mediated immunosuppression while preserving beneficial metabolic functions.

Moreover, while targeting GABA receptors especially GABA_A_ receptor subtype is considered a potential therapeutic strategy, it faces significant challenges. A primary contradiction is that many receptor subtypes are expressed in both the central nervous system (CNS) and peripheral tissues, requiring drug development to balance efficacy and neurotoxicity risks. Several GABA receptor modulators are already in clinical use for neurological conditions, including benzodiazepines as GABA_A_ receptor positive allosteric modulators, baclofen as a GABA_B_ receptor agonist, and gabapentinoids, offering a potential starting point for drug repurposing strategies in oncology. However, drugs targeting these receptors must either achieve peripheral restriction or carefully balance efficacy against central nervous system penetration [90]. Emerging approaches include developing peripherally restricted GABA receptor antagonists that do not cross the blood-brain barrier, exploiting subtype-specific expression patterns such as GABRP in ovarian [91] and breast cancer [92] and GABRD in colorectal cancer [82], and using antibody-drug conjugates or nanoparticle delivery systems to achieve tumor-specific targeting [61]. For example, the GABRP subtype, highly expressed in peripheral tissues, may serve as a safer target, but its universality and efficacy across different cancers still need extensive validation. Targeting GABA metabolic enzymes or receptors offers potential for reversing immunosuppression and developing new combination immunotherapy strategies. Emerging evidence from mouse models demonstrates that combining GABA pathway inhibitors with anti-PD-1 immune checkpoint blockade therapy can synergistically enhance anti-tumor immunity, leading to reduced tumor burden and improved survival compared to either monotherapy [17]. Future research should explore the heterogeneity of GABA immunomodulatory roles across cancer types and create specific delivery systems to minimize neurotoxicity while maximizing therapeutic benefit.

Finally, it is noteworthy that as a key regulatory molecule of the gut-brain axis, the in vivo regulatory role of GABA in tumor progression requires further verification through targeted animal models. For instance, mice with abnormal GABA secretion, such as depressive model mice, can be used as research subjects. After implanting gastric cancer cells into these mice, systematic observations can be made on the biological behavioral changes of gastric cancer cells, including malignant proliferation and metastasis. Relevant research directions have been covered in the latest studies; additionally, direct intracerebral injection of GABA can be performed to clarify its specific impact on the occurrence and development of gastric cancer, thereby providing in vivo experimental evidence for revealing the GABA-mediated gut-brain axis-tumor regulatory mechanism.

In summary, future research should focus on clarifying the expression contradictions of key GABA metabolic enzymes, further dissecting the molecular basis of receptor subtype-specific functions, elucidating the interactive relationships between multiple immunosuppressive mechanisms, mapping a more refined atlas of TME metabolic regulatory networks, and developing precise targeted strategies based on these advancements.

## Declaration of generative AI use

During the preparation of this work the authors used Doubao AI in order to refine grammatical errors and conduct spelling checks. After using this tool, the authors reviewed and edited the content as needed and takes full responsibility for the content of the published article.

## CRediT authorship contribution statement

**Yujie Wang:** Data curation, Writing – original draft. **Junzhao Shao:** Data curation, Writing – original draft. **Ming Wang:** Writing – original draft. **Zhenlong Sun:** Writing – original draft. **Nan Wang:** Formal analysis, Writing – original draft. **Wei Li:** Conceptualization, Supervision, Writing – review & editing. **Hong Luo:** Supervision, Writing – review & editing.

## Funding

This paper is supported by the Shandong Provincial Natural Science Foundation (ZR2024QH297) and the Shandong Provincial Medical and Health Science and Technology Development Plan Project (20231101195).

## Declaration of competing interest

The authors declare that they have no known competing financial interests or personal relationships that could have appeared to influence the work reported in this paper.

## Data Availability

Raw mRNA sequencing count data of gastric tumors in Fig. 3 were obtained from The Cancer Genome Atlas (TCGA) via the UCSC Xena Data Portal (http://xena.ucsc.edu/). To mitigate the skewness of count distributions and stabilize variance across samples, gene expression levels were transformed using a log (counts+1) normalization. This transformation preserves the relative differences between samples while reducing the influence of extremely high counts, and is commonly applied in downstream differential expression and correlation analyses. After retaining samples with both RNA-seq data and clinical information, we ultimately selected 448 samples for further analysis (36 in the control group and 423 tumor samples). For statistical analysis, an unpaired *t*-test was employed to compare the means between the two independent groups.
